# RETRACTION: Omega‐3 Fatty Acids Inhibit Tumor Growth in a Rat Model of Bladder Cancer

**DOI:** 10.1155/bmri/9864535

**Published:** 2026-06-11

**Authors:** BioMed Research International

RETRACTION: B. Parada, F. Reis, R. Cerejo, P. Garrido, J. Sereno, M. Xavier‐Cunha, P. Neto, A. Mota, A. Figueiredo, F. Teixeira, “Omega‐3 Fatty Acids Inhibit Tumor Growth in a Rat Model of Bladder Cancer,” *BioMed Research International*, 2013, 368178, https://doi.org/10.1155/2013/368178

The above article, published online on 20 June 2013 in Wiley Online Library (wileyonlinelibrary.com/), has been retracted by agreement between the journal Editor‐in‐Chief, Yoel Klug; and John Wiley & Sons Ltd. The retraction has been agreed due to concerns identified with the figures, both directly and on PubPeer [1]. Further investigation by the Publisher noted additional concerns. To summarize:

• In Figure 1, Panel C is identical to Panel C in Figure 1 of [2]
• In Figure 2, Panel B appears identical to:
  o. Panel B in Figure 3 of [3]
  o. Panel A in Figure 2 of [2], after rotation
  o. Panel B in Figure 2 of [4]
• In Figure 2, Panel C2 appears identical to Panel C2 In Figure 2 of [4]

After assessment of the concerns and the authors’ response by the editorial board, the results and conclusions can no longer be considered reliable, and the article is therefore retracted.

The authors disagree with the retraction.
